# Understanding the Seasonal Effect of Metabolite Production in *Terminalia catappa* L. Leaves through a Concatenated MS- and NMR-Based Metabolomics Approach

**DOI:** 10.3390/metabo13030349

**Published:** 2023-02-27

**Authors:** Ana C. Zanatta, Natália Carolina Vieira, Renato Dantas-Medeiros, Wagner Vilegas, RuAngelie Edrada-Ebel

**Affiliations:** 1 Department of Biochemistry and Organic Chemistry, São Paulo State University (UNESP), Araraquara 14800-060, SP, Brazil; 2 Institute of Biosciences, São Paulo State University (UNESP), São Vicente 11380-972, SP, Brazil; 3 Strathclyde Institute of Pharmacy and Biomedical Sciences, University of Strathclyde, Glasgow G4 0RE, UK; 4 Department of Pharmaceutical Sciences, Faculty of Pharmacy, Federal University of Rio Grande do Norte, Natal 59012-570, RN, Brazil

**Keywords:** plant metabolomics, specialized metabolites, data fusion, seasonality

## Abstract

*Terminalia catappa* L. (Combretaceae) is a medicinal plant that is part of the Brazilian biodiversity; this plant is popularly used for the treatment of a wide range of diseases. To better understand the chemical composition of *T. catappa* in different seasons, we conducted a thorough study using LC-MS and NMR data analysis techniques. The study helped obtain a chemical profile of the plant ethanolic extracts in different seasons of the year (spring, summer, autumn, and winter). The dereplication of LC-HRMS data allowed the annotation of 90 compounds in the extracts of *T. catappa* (hydrolyzable tannins, ellagic acid derivatives, and glycosylated flavonoids). Triterpenes and *C*-glycosyl flavones were the compounds that significantly contributed to differences observed between *T. catappa* plant samples harvested in autumn/winter and spring, respectively. The variations observed in the compound composition of the plant leaves may be related to processes induced by environmental stress and leaf development. Data fusion applied in the metabolomic profiling study allowed us to identify metabolites with greater confidence, and provided a better understanding regarding the production of specialized metabolites in *T. catappa* leaves under different environmental conditions, which may be useful to establish appropriate quality criteria for the standardization of this medicinal plant.

## 1. Introduction

Brazil is one of the few countries in the world with incredibly rich biodiversity. By virtue of this enormous biodiversity, Brazilian medicinal plants have become the focus of chemical studies among researchers mainly due to the huge variety of compounds (both discovered and “yet to be” discovered) present in these plants that are found to be useful for the treatment of a wide range of diseases in humans [1,2]. The study of medicinal plants in Brazil will help us identify new compounds present in unexplored plant matrices, and this can pave the way for the discovery of new, useful secondary metabolites [3,4].

*Terminalia catappa* species can be found in all the regions of Brazil, and especially in the Southeastern region of the country due to the warm weather in this area, which makes it suitable for the development and growth of the plant. The *T. catappa* plant is a halophyte species and is prevalent in both the tropical and subtropical regions of Brazil, and most particularly in coastal areas, where they are known for their extensive shades along the beachfront of the country’s beaches [5,6,7]. This plant is widely popular among the Brazilian populace, who often use a variety of names, including “amendoeira-da-praia”, “cuca”, and “chapéu de sol”, to refer to the plant [8]. Ayurvedic medicine uses species of the *Terminalia* genus for the treatment of abdominal and back pains, cough, cold, conjunctivitis, diarrhea and dysentery, inflammation, and ulcers, among other diseases [9]. Pharmacological studies of *T. catappa* have shown that the ethanolic extracts of the plant present potential gastroprotective, anti-inflammatory, and nontoxicity properties in the acute models evaluated and reported in the literature [10,11]. In addition, *T. catappa* has also been reported to possess other relevant pharmacological properties [9]; these include antibacterial and antifungal [12,13,14], antioxidative [15,16], anti-inflammatory [17,18], hepatoprotective [16], and antidiabetic properties [19,20], in addition to carcinogenesis-preventing effects [21,22], antimalarial [23,24], and antinociceptive [25] properties. These properties evidently point to the promising medicinal potential of this plant in terms of application for the treatment of a wide range of diseases.

Previous phytochemical studies conducted on the extracts of *T. catappa* leaves have shown that the species possesses mainly compounds of the phenolic classes [11,26,27], including flavonoids [26,27,28] and hydrolyzable tannins [11,27]. Diverse classes of metabolites found in *T. catappa*, which are known for their benefits to human health, may also play important ecological roles. However, there is still a scarcity of knowledge regarding the relationship between the chemical composition of the species and its metabolic variation when it is harvested at different times of the year, which is an essential step previous to the use of the plant as a pharmaceutical agent. Remarkably, seasonality is one of the main abiotic factors that directly influence the response of the biosynthetic mechanism in the plant; this is because specialized metabolites can act as mediators of plant–environment interactions, and consequently, the synthesis and accumulation of these metabolites can be affected by external environmental cues [29]. Thus, seasonal studies are of great importance to determine the influence of factors, such as season, as well as time and day of harvesting, on qualitative and quantitative variations in the composition of secondary metabolism, thus helping to ensure quality control and therefore safety and efficacy of herbal products [30,31].

Metabolomics is considered one of the most highly promising techniques for determining different profiles of metabolites in medicinal plants and the relationship between the composition of the metabolites and the surrounding environment [32]. Among the analytical techniques applied for determining the profiles of metabolites, nuclear magnetic resonance (NMR) and high-resolution mass spectrometry (HRMS) have become widely popular among researchers in the last few years [33]. The NMR technique provides quantitative data and has the advantage of being a highly reproducible and noninvasive method, while HRMS is more sensitive and has the ability to measure a larger number of molecules in complex matrices [33,34,35]. Thus, due to the complementary application advantages of the two techniques, several studies reported in the literature have employed NMR in combination with HRMS for metabolomics analyses, where they obtained highly satisfactory results [35]. Furthermore, multivariate analysis methods (e.g., principal component analysis (PCA), partial least squares discriminant analysis (PLS-DA, and/or orthogonal least squares discriminant analysis (OPLS-DA)) are often used to help interpret the large amount of data generated [36].

Previous studies reported in the literature have successfully employed data fusion based on MS and NMR techniques for the conduct of metabolomics analyses [37,38,39,40]; these studies have shown that the application of fused data for metabolomics analyses leads to more robust results, and allows one to annotate/identify the compounds with a high level of confidence; additionally, the MS/NMR-based data fusion technique is found to be quite useful when it comes to further interpretation of analytical results.

In this study, we tested the hypothesis that environmental factors associated with seasonal variations exert influence on the qualitative and quantitative production of specialized metabolites in *T. catappa* leaves and, therefore, may affect the concentration of active components present in the medicinal plant. For this evaluation, the present work employed the metabolomics approach based on the application of advanced analytical techniques (UHPLC-ESI-HRMS and NMR) and multivariate statistical analyses in order to evaluate the role played by a set of variables related to chemical composition and the environment, with a view to improving our understanding regarding seasonal effects on the metabolites of *T. catappa* leaves, as well as establishing a metabolic fingerprinting for the quality control of this species.

## 2. Materials and Methods

### 2.1. Chemicals and Reagents

Ethanol (HPLC grade; Tedia^®^, Fairfield, OH, USA) was used in the extraction procedure, while methanol, formic acid (LC-MS grade; Merck^®^, Darmstadt, Germany), and ultrapurified water (Millipore^®^, Burlington, MA, USA) were used as mobile phase components.

### 2.2. Plant Material

*T. catappa* leaves were collected from the beachfront of the city of Santos, São Paulo, Brazil, located at latitude 23°58′13′′ S, longitude 46°19′53′′ W, and altitude 17 m. The harvesting of the plant species was carried out during the summer (February/2017 (Tc1) and February/2018 (Tc7)), autumn (May/2017 (Tc2), June/2017 (Tc3), April/2018 (Tc8), and June/2018 (Tc9)), winter (August/2017 (Tc4), July/2018 (Tc10), and August/2018 (Tc11)), and spring (October/2017 (Tc5), December/2017 (Tc6), October/2018 (Tc12), and November/2018 (Tc13)) seasons over a two-year period (2017 and 2018); this was done in order to evaluate seasonal variability of secondary metabolites in the plant species. For this study, leaves were harvested with the help of a pole pruner and carried out with at least three independent replicates for each season, each composed of 5 individuals at an adult stage. The average values for height and diameter at breast height (DBH) of the trees are 10.0 m and 0.50 m, respectively. The data collected were added to the SisGen platform (National System of Management of Genetic Heritage and Associated Traditional Knowledge) as genetic patrimony, with registration number A3476AF.

For the transfer of the materials from São Paulo State University (UNESP), Brazil, to the University of Strathclyde, Scotland; all samples were prepared in accordance with the Brazilian laws for access and shipment of genetic heritage material. The R0418CB shipment number was issued by SisGen, under the authorization of the Genetic Heritage Management Council (CCGEN).

### 2.3. Climate Data

Meteorological data for the plant harvest months in Santos-SP over the years 2017 and 2018 were provided by the Meteorological Data Storage Section (SADMET) of the National Institute of Meteorology (INMET) and are provided in Supplementary Table S1 (average temperature (°C), solar radiation (kJ/m^2^), air humidity (%) and rainfall (mm)).

### 2.4. Ethanolic Extract and Samples Preparation

The plant material was washed and dried in an oven with air circulation at 40 °C, and the dried material was ground in an analytical mill (model IKA A11 basic). A total mass of 100 mg of powder was extracted with 1 mL of EtOH in an ultrasound bath, three times for 20 min. After that, the resulting material was centrifuged at 13,000 rpm, and the supernatant was filtered through a Millex^®^ PTFE filter (0.22 µm, 25mm). The organic solvent was dried in N_2_ atmosphere. 

For NMR analyses, samples were prepared by dissolving each extract in 650 µL of DMSO-*d*_6_ (Sigma Aldrich^®^, St. Louis, MO, USA) to obtain the concentration of 5 mg/mL, then transferred to 5 mm 7″ NMR tubes. As for the LC-MS analysis, the extracts were suspended in methanol at a concentration of 1.0 mg/mL and filtered through a Millex^®^ PTFE filter with a pore size of 0.22 µm. 

### 2.5. NMR Analysis

NMR experiments were performed using a Bruker^®^ AVIII HD 500 (11.7 T). Data acquisition for two-dimensional (2D) ^1^H–^1^H *J*-resolved (*J*-res) followed the parameters used in the previous work developed by Zanatta et al. [37]. Briefly, the following acquisition parameters were used: 32 scans and 64 increments per scan, data point width of 3.56 kHz for F2 (chemical shift axis), 50 Hz for F1 (spin–spin coupling constant axis), and application of the selective presaturation method for solvent signal suppression.

### 2.6. UHPLC-ESI-HRMS Analysis

Samples were analyzed on an ultra-high-performance liquid chromatography analytical system (UHPLC) (Accela, Thermo Fisher Scientific^®^, Bremen, Germany) coupled to a high-resolution Exactive-Orbitrap mass spectrometer (Thermo Fisher Scientific^®^, Bremen, Germany). Data acquisition was performed as described by Zanatta et al. [37]. In summary, the following analysis conditions were used for sample elution: C-18 column (ACE, 75 mm, id 3.0 mm, 5 µm); injection volume of 10 µL; flow rate of 300 µL/min; mobile phase consisting of water (solvent A) and methanol (solvent B) both acidified with 0.1% formic acid, ramped from 5 to 100% (B) for 45 min; and for mass spectrometry analyses: acquisition range of *m*/*z* 150–2000, in negative and positive ionization modes; spray voltage of 4.5 kV for the positive mode and 4.0 kV for the negative mode; and capillary temperature of 280 °C. The mass accuracy was set to less than 3.0 ppm. The Orbitrap mass analyzer can limit the mass error within ± 3.0 ppm. The instrument was calibrated to maintain a mass accuracy of ± 1.0 ppm by applying the lock mass function. The instrument was externally calibrated according to the manufacturer’s instructions before the run and was internally calibrated during the run using lock masses. In positive ion mode, lock masses were m/z 83.06037 (acetonitrile dimer) and *m*/*z* 195.08625 (caffeine), and in negative ion mode, the lock mass was *m*/*z* 91.00368 (formic acid dimer). The samples were run randomly, with solvent blanks and an external standard analyzed at the beginning and end of the sequence, as well as every after 10 samples. Retention time migration was quality controlled (QC) by checking against the external standard reserpine eluting at 11.38 ± 0.2 min and *m*/*z* of 609.2794 [M+H]^+^ that was validated during the gap-filling step when processing the spectral dataset by MZmine2 (further described under Section 2.7), while peak alignment assigned an equal weight of importance to the retention time and *m*/*z* data replicates [41,42]. Chromatographic peaks along with their corresponding spectral data were validated using an Excel macro to QC peak signals to noise against the blank and within replicates [41,42]. The Xcalibur software (version 3.0, Thermo Finnigan LLC, San Jose, CA, USA) was used to acquire and process the chromatographic and spectral data.

### 2.7. Data Processing

The data obtained after analysis of the extracts by *J*-res NMR and UHPLC-ESI-HRMS were processed for further annotation and multivariate analysis steps. *J*-res spectra were processed using MestReNova x64 software (version 14.1.2, Mestrelab Research SL, Santiago de Compostela, Spain), and the following processing steps were performed: T1 noise reduction, 45° tilt, and symmetrization by *J*-res sensitivity enhancement. The data were exported as the one-dimensional projection (F2 axis) of the two-dimensional *J*-res spectra. The projection spectra were stacked and chemical shift (*δ*) values set between 0.0 and 9.0 ppm. They were then prepared for output using a bin width of 0.04 ppm and the average sum for bin intensities.

For LC-MS data processing, raw files obtained in the two ionization modes ([M+H]^+^ and [M-H]^−^) were first converted in MSConvert software (version 3, ProteoWizard) to mzML format and then processed in MZmine2 v. 2.53 (http://mzmine.sourceforge.net/, accessed on 20 December 2023) [43,44]; the following processing steps were used: mass detection (MS1 noise level of 1.0 × 10^3^); chromatogram builder (minimum time span of 0.2 min; minimum height of 1.0 × 10^4^, and mass tolerance of 0.001 *m*/*z* or 5.0 ppm); chromatogram deconvolution (algorithm local minimum search; chromatographic threshold of 5%; search minimum in RT range of 0.4 min; minimum relative height of 5%; minimum absolute height of 1.0 × 10^4^; min ratio of peak top/edge of 3; and peak duration range of 0.2–5 min); deisotoping; filtering; alignment; gap filling using the peak finder algorithm. After gap filling, all peaks found in solvent blanks were deleted. Additionally, the steps of adduct identification, peak complex search, and molecular formula prediction were performed, with the latter using given elemental and heuristic constraints [45]. At last, a feature table was generated containing information on peak areas, exact mass, and molecular formula for all samples. To perform peak annotation, the exported table was analyzed in an Excel macro containing the Dictionary of Natural Products database [41,46]. Hits were considered as true by comparison with data reported in the literature for the genus and/or family of the *T. catappa* plant.

The processed NMR and MS output data were then concatenated in order to accelerate the annotation of the specialized metabolites associated with the seasonal variabilities of each sample; the data were separated into two blocks (*J*-res data block and LC-MS data block), which were organized in an Excel^®^ spreadsheet and scaled according to Equation (1) as follows:

(1) $${\hat{x}}_{n}=\frac{x_{n}}{\sum \sigma_{block}}$$

where $\hat{x}$ is the estimated value for each variable (the residuals), x is the observed variable (peak intensity and peak area for NMR and LC-MS and data, respectively), n is the number of values in the dataset, and $\sigma_{block}$ is the standard deviation of each block [37,38].

### 2.8. Multivariate Data Analysis

The data processed by both techniques (NMR and LC-MS) separately and MS-NMR fused were submitted to SIMCA-P v. 17.0 software (Umetrics^®^, Umeå, Sweden) multivariate data analysis (MVDA) to investigate possible correlations between *T. catappa* harvests. For the analysis, the identifiers were specified as follows: chemical variables as primary variable IDs, environmental variables as secondary observation IDs, and seasons as class ID specification. The Pareto algorithm was used to scale the primary variables for the three data sets (NMR, MS, and MS-NMR fused data). For the fused data, block-wise scaling was applied, allowing each block of variables (NMR and MS) to be considered as a unit and given the appropriate variation. In order to evaluate the interaction that certain variables (compounds) have on the clusters, supervised analyses such as partial least squares discriminant analysis regression (PLS-DA) and orthogonal partial least squares discriminant analysis (OPLS-DA) were performed. The most distinct season was statistically discriminated and compared to the other seasons using OPLS-DA analysis. Permutation tests (n = 100 permutations) were conducted to check the validity and degree of overfitting for the PLS and OPLS models using both MS and MS-NMR fused datasets.

As PLS and OPLS models are quite complex and have many components and a multiplicity of responses, the parameter variable importance on projection (VIP), which summarizes the importance of chemical variables, was used for interpretative clarity. VIPs with false discovery rate (FDR) values ≤0.05 were identified as discriminant metabolites. In calculating the FDR, the equation from Benjamini–Hochberg [47] was used. The discriminant features with *p* ≤ 0.05 were ranked from smallest to largest. Rankings for each discriminant were accordingly assigned, with the smallest *p*-value ranked 1, the next smallest ranked 2, and so on. Thus, to calculate the FDR value for each respective *p*-values, the following formula was used: FDR = (*i*/*m*) × Q, where: *i* = rank of the *p*-value; *m* = the total number of ranked discriminants with *p* ≤ 0.05, Q = acceptable %FDR at 5%.

Box-and-whisker plots were generated using GraphPad Prism 8.4.3 software (GraphPad Software, San Diego, CA, USA), which were plotted from the normalized data of the variables assigned to the discriminating metabolites. This analysis was performed using one-way ANOVA followed by Tukey’s test. All results are presented as mean ± standard deviation (SD).

## 3. Results

### 3.1. Metabolite Profiling

The extracts prepared from the seasonal harvests of the Brazilian *T. catappa* leaves exhibited comparable metabolic profiles when evaluated by NMR and LC-HRMS techniques. Supplementary Figures S1 and S2 (see the Supplementary Materials) show the 1D *J*-res NMR projection spectra and 2D *J*-res NMR spectra, and Supplementary Figure S3 shows the representative LC-HRMS chromatograms related to the negative and positive ionization modes obtained from the preprocessing procedure using the MZmine2 software [43] for each extract from the harvested plant species.

The spectral profile of the *T. catappa* harvest extracts obtained from NMR analysis exhibited signals mainly in the following regions: 0.50–2.00 ppm (aliphatic proton shifts), 3.00–6.00 ppm (sugar and organic acids proton shifts), and 6.00–9.00 ppm (aromatic proton shifts) (Supplementary Figures S1 and S2).

Following the LC-ESI-HRMS data search in the macro compound identification library, which contains the Dictionary of Natural Products (DNP), a diversity of chemical structures and classes of representative compounds or chemical markers of the genus and/or family of the *T. catappa* plant were tentatively dereplicated; this analysis allowed us to identify 90 specialized metabolites in the extracts of *T. catappa*. The application of a high-resolution device along with the MZmine2 preprocessing tool helped predict the mass and molecular formula of each compound with a high degree of accuracy for each of the compounds annotated; these annotations are Level 3, as they combine the match precursor *m*/*z* to a metabolite database [48].

A thorough analysis of the *T. catappa* leaves showed that this plant has mostly in its composition hydrolyzable tannins, including gallotannins (e.g., *m*/*z* 331.0679 [M-H]^−^, *m*/*z* 483.0793 [M-H]^−^, *m*/*z* 637.1035 [M+H]^+^, *m*/*z* 789.1152 [M+H]^+^ and *m*/*z* 939.1133 [M-H]^−^) and ellagitannins (e.g., *m*/*z* 633.0753 [M-H]^−^, *m*/*z* 781.0549 [M-H]^−^, *m*/*z* 785.0874 [M-H]^−^, *m*/*z* 953.0931 [M-H]^−^, *m*/*z* 1083.0620 [M-H]^−^ and *m*/*z* 1085.0738 [M-H]^−^), ellagic acid derivatives (e.g., *m*/*z* 300.9996 [M-H]^−^, *m*/*z* 315.0156 [M-H]^−^, *m*/*z* 329.0311 [M-H]^−^, *m*/*z* 345.0604 [M-H]^−^, *m*/*z* 447.0581 [M-H]^−^ and *m*/*z* 461.0741 [M-H]^−^), ellagic acid glycosides (e.g., *m*/*z* 447.0581 [M-H]^−^, *m*/*z* 461.0741 [M-H]^−^ and *m*/*z* 601.0826 [M+H]^+^) and glycosylated flavonoids (e.g., *m*/*z* 433.1129 [M+H]^+^, *m*/*z* 449.1077 [M+H]^+^, *m*/*z* 465.1027 [M+H]^+^, *m*/*z* 595.1446 [M+H]^+^ and *m*/*z* 611.1605 [M+H]^+^), galloyl flavonoids (e.g., *m*/*z* 585.1239 [M+H]^+^ and *m*/*z* 601.1186 [M+H]^+^), triterpenes (e.g., *m*/*z* 453.3362 [M+H]^+^, *m*/*z* 457.3679 [M+H]^+^, *m*/*z* 473.3629 [M+H]^+^, *m*/*z* 489.3575 [M+H]^+^, *m*/*z* 505.3525 [M+H]^+^, and *m*/*z* 521.3476 [M+H]^+^), among other compounds. A comprehensive annotation/identification of the compounds present in the *T. catappa* harvest extracts can be found in Supplementary Table S2.

### 3.2. Seasonality Assessment from MS-NMR Fused Data

**Figure 1:**
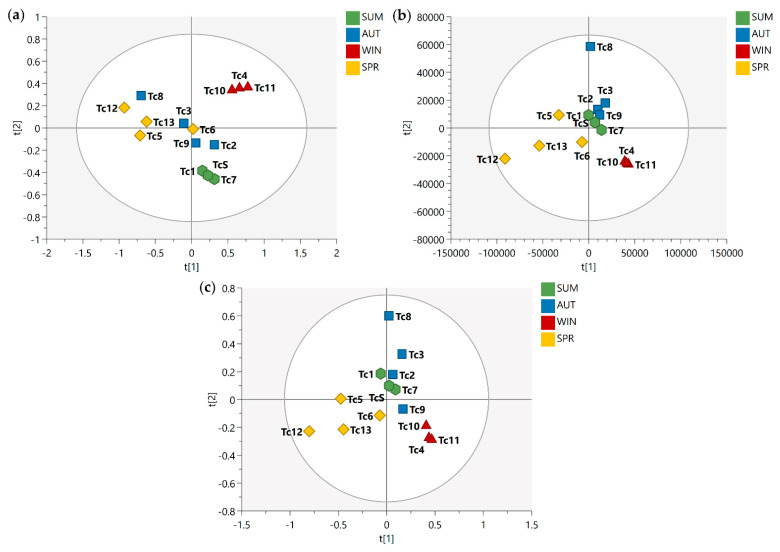
PLS-DA score scatter plots of the *T. catappa* harvest leaves obtained: (**a**) NMR; (**b**) MS; (**c**) MS-NMR fused analysis for Summer (SUM), Autumn (AUT), Winter (WIN), and Spring (SPR) samples. Season samples are represented by hexagon—SUM (Tc1, Tc7, and TcS), square—AUT (Tc2, Tc3, Tc8, and Tc9), triangle—WIN (Tc4, Tc10, and Tc11), and diamond—SPR (Tc5, Tc6, Tc12, and Tc13).

After the conduct of qualitative analysis of the metabolic profile of *T. catappa* harvest leaves, supervised analyses (PLS-DA and OPLS-DA) were performed in order to evaluate the impact of some variables (metabolites) on the seasonal samples. Figure 1 shows the PLS-DA score scatter plot for 1D *J*-res NMR, MS, and MS-NMR fused data.

Looking at the PLS-DA score scatter plot from the NMR data (Figure 1a), the separation of the samples into distinct groups could be observed particularly between the summer and winter samples. Interestingly, there is an overlap between the samples harvested in the spring and autumn. In the MS data plot (Figure 1b), a slightly different result was obtained; there is a noticeable isolation of the samples harvested in the winter, while the summer samples show a different distribution, being arranged between the spring and autumn samples. Finally, with the concatenation of the NMR and MS data (Figure 1c), one will observe that the autumn samples exhibit intermediate characteristics between the summer and winter samples, while a sloping trend is observed from the spring samples to the winter samples.

**Figure 2:**
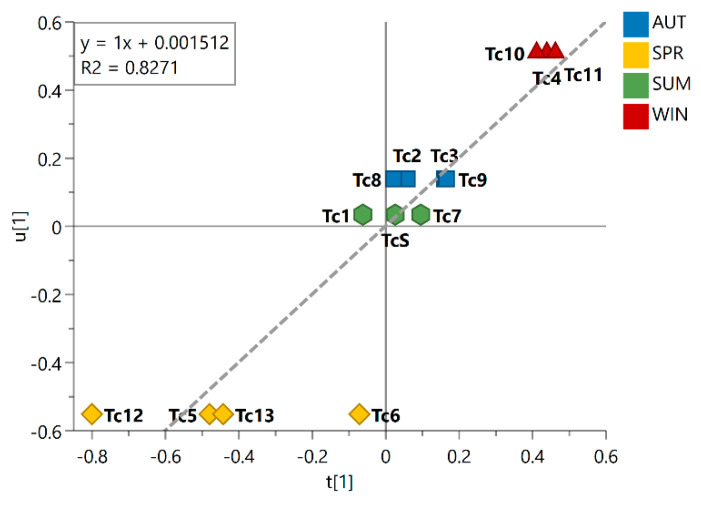
Inner relation plot of the PLS-DA model for season groups. The line in the plot shows the linear relationship between the samples following Spring (SPR), Summer (SUM), Autumn (AUT), and Winter (WIN). Linear regression equation and coefficient of determination R^2^ are shown in the graph. Season samples are represented by hexagon—SUM (Tc1, Tc7, and TcS), square—AUT (Tc2, Tc3, Tc8, and Tc9), triangle—WIN (Tc4, Tc10, and Tc11), and diamond—SPR (Tc5, Tc6, Tc12, and Tc13).

This trend is evident in the inner relation plot (Figure 2), which showed a linear relationship between the harvested samples that is consistent with the expected seasonal variations, spring → summer → autumn → winter. Looking at this inner relation plot, it could also be observed that the spring samples lie farthest from the other seasonal groups, while the summer and autumn samples lie at the closest distance to each other; this finding points to the most distinct season and the most similar seasons, respectively, among the seasonal groups investigated. Additionally, the dispersive behavior observed for the spring samples indicated great chemical variability within the group. As observed in the PLS-DA score plot, the summer samples are clustered around the origin, indicating that the chemical variation in this group showed no statistically significant contribution to the differentiation of the seasonal groups. The obtained R^2^ value of 0.8271 illustrated the goodness of fit of the model for the different seasonal samples.

The permutation test for PLS-DA was used for the validation of the model (Supplementary Figure S4). The plots obtained from this test showed that the R^2^ values are greater than those of Q^2^, and the Q^2^ regression line exhibited a negative value for intercept, which showed that the PLS-DA models are valid and not overfitted, and do exhibit a good degree of predictability; synonymously, this model can be used to predict seasonal biomarkers present in the plant extract.

**Figure 3:**
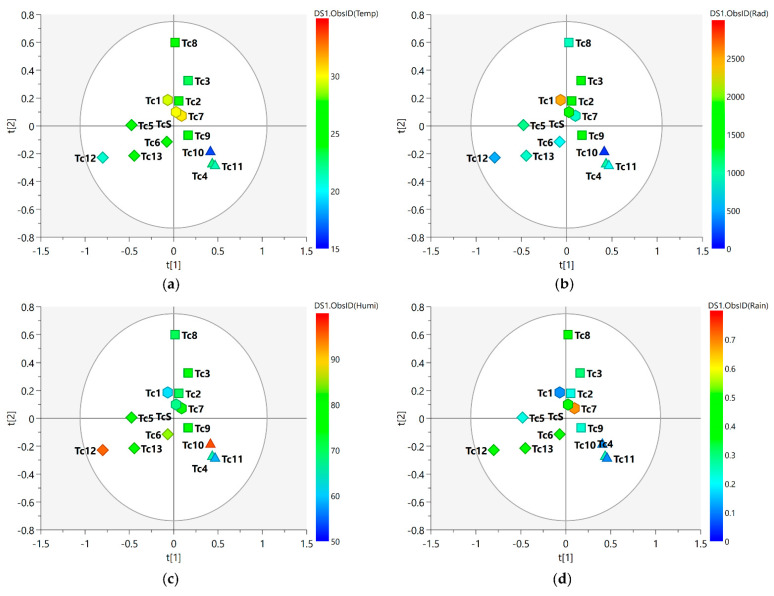
PLS-DA score scatter plots of the MS-NMR fused data of *T. catappa* seasonal harvests and the correlation with the environmental factors. Samples are categorized according to the values of observations for environmental factors: (**a**) Temp = temperature (°C); (**b**) Rad = Solar radiation (kJ/m^2^); (**c**) Humi = relative humidity (%); (**d**) Rain = rainfall (mm). Season samples are represented by hexagon—Summer (Tc1, Tc7, and TcS), square—Autumn (Tc2, Tc3, Tc8, and Tc9), triangle—Winter (Tc4, Tc10, and Tc11), and diamond—Spring (Tc5, Tc6, Tc12, and Tc13). Blue indicates minimum values; red indicates maximum values.

Figure 3 shows the MS-NMR fused data for the seasonal harvest leaves where the values for environmental factors (values for temperature, solar radiation, relative humidity, and rainfall are presented in Supplementary Table S1) are employed in distinct colors.

Considering the meteorological conditions of the area in which the plant was harvested, one will observe that these environmental factors present punctual variations throughout the year. The spring (Tc5, Tc6, Tc12, and Tc13) and autumn (Tc2, Tc3, Tc8, and Tc9) samples exhibited the greatest dispersion, and this translated into a relatively wider variation in chemical composition within the group; on the other hand, the tight clustering observed for the summer (Tc1, Tc7, and TcS) and winter samples (Tc4, Tc10, and Tc11) may be due to the little variation observed in their chemical profiles. With regard to the spring samples, the slightly higher average values of humidity (Figure 3c) and rainfall (Figure 3d) may have been a contributing factor to the distinct characteristics observed for the samples harvested during this season. With regard to the separation of the samples, one will notice that the spring and winter samples are positioned in distinct groups; this points to greater differences between the two seasonal groups. This difference in position can be explained by the amount of rainfall during the period (Figure 3d), with higher values in spring and lower values in winter; this variation in the amount of rainfall may explain the differences observed in the chemical profile of these samples.

**Figure 4:**
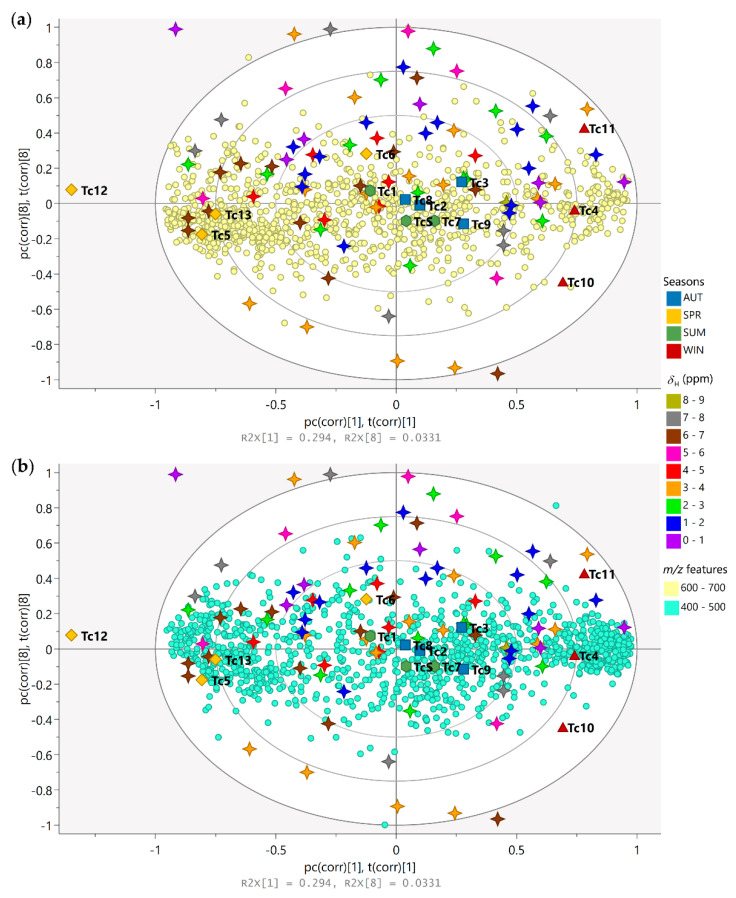
Biplot (loadings p and scores t) based on PLS-DA model describing the simultaneous correlation between the chemical variables (*m*/*z* features and ^1^H NMR chemical shifts) and the grouping of samples (seasonal harvests). Season samples are represented by hexagon—Summer (SUM: Tc1, Tc7, and TcS), square—Autumn (AUT: Tc2, Tc3, Tc8, and Tc9), triangle—Winter (WIN: Tc4, Tc10, and Tc11), and diamond—Spring (SPR: Tc5, Tc6, Tc12, and Tc13); *m*/*z* features by circles ((**a**) 400 to 500 and (**b**) 600 to 700 Da); ^1^H NMR chemical shifts (*δ*) by 4-point stars (0.0 to 9.0 ppm).

The biplot constructed based on the PLS-DA model was used for the analysis of the correlation and the simultaneous interpretation of the chemical variables, considering the grouping of the samples (Figure 4). The spring samples on the left quadrants (Tc5 and Tc13) lie close to signals with chemical shifts between *δ*_H_ 5.00 and 7.00 (stars colored in pink and brown, respectively), which are attributed to protons in the aromatic region, and characteristics with a higher density of features with molecular weights ranging from 600 to 700 Da (Figure 4a); these observations showed that the variables are in greater quantity in these samples and in lesser quantity in the winter samples (right quadrants). On the right quadrants of the plot, the winter samples (Tc4, Tc10, and Tc11) can be found lying close to signals with chemical shifts between *δ*_H_ 0.00–2.00 (stars colored in purple and blue, respectively) and *δ*_H_ 3.00–4.00 (stars colored in orange), which are attributed to protons in the aliphatic region, in combination with compounds with a higher density of features with molecular weights ranging from 400 to 600 Da (Figure 4b). Another observation that can be made from the biplot is that the summer and autumn samples near the plot origin (zero axis) have average properties, while the variables nearer to the zero axis do not play any significant role in the differentiation of the samples.

**Figure 5:**
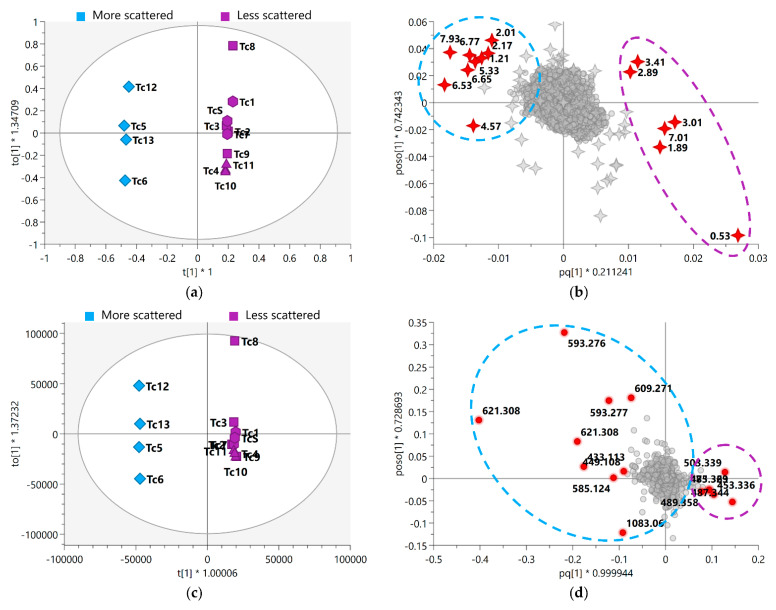
OPLS-DA score scatter plot of (**a**) MS-NMR fused data and (**c**) MS data with samples categorized according to the harvest season; OPLS-DA shows the variation in chemical composition within groups (vertical dimension) and between groups (horizontal dimension). OPLS-DA loadings plot with highlighted variables of the 15 VIP features of (**b**) MS-NMR fused data and (**d**) MS data. More scattered samples: Spring (Tc5, Tc6, Tc12 and Tc13); Less scattered samples: Summer (Tc1, Tc7, and TcS); Autumn (Tc2, Tc3, Tc8, and Tc9); Winter (Tc4, Tc10, and Tc11). Season samples are represented by hexagon—Summer, square—Autumn, triangle—Winter, and diamond—Spring, *m*/*z* features by circles, and ^1^H NMR chemical shifts (*δ*) by 4-point stars. Annotation of the highlighted discriminating metabolites is provided in Table 1 and Table 2. Where * means multiplication sign.

OPLS-DA analysis was performed to improve the visualization and interpretation of the model, and they were found to have good fit and a satisfactory degree of predictability (Supplementary Figure S5). In this model, the seasonal samples were separated into relatively scattered (spring) and more clustered (autumn, summer, and winter) groups (Figure 5). The resulting model related to the fused MS-NMR data exhibited a variation of 21.1% (R2X(1) = 0.211) between groups and a variation of 22.2% (R2X(o1) = 0.222) within the groups; with regard to the MS data, the model exhibited variations of 23.2% (R2X(1) = 0.232) and 28.0% (R2X(o1) = 0.280) between and within the groups, respectively.

The loadings plot (Figure 5b,d) shows the variables responsible for the differences in the grouping of the seasonal samples. In addition, the features were ranked according to their significance to the model by VIP score; here, 15 variables with the highest VIPs related to both the MS-NMR fused data (Figure 5b) and MS data (Figure 5d) are worthy of attention (red circle). Based on these variables, the FDR values were calculated; these values represented the true-positive annotation and indicated the discriminating metabolites associated with the seasonal harvests. The discriminant features for each analysis (MS-NMR fused data and MS data) and the proposed metabolite annotation are presented in Table 1 and Table 2.

**Figure 6:**
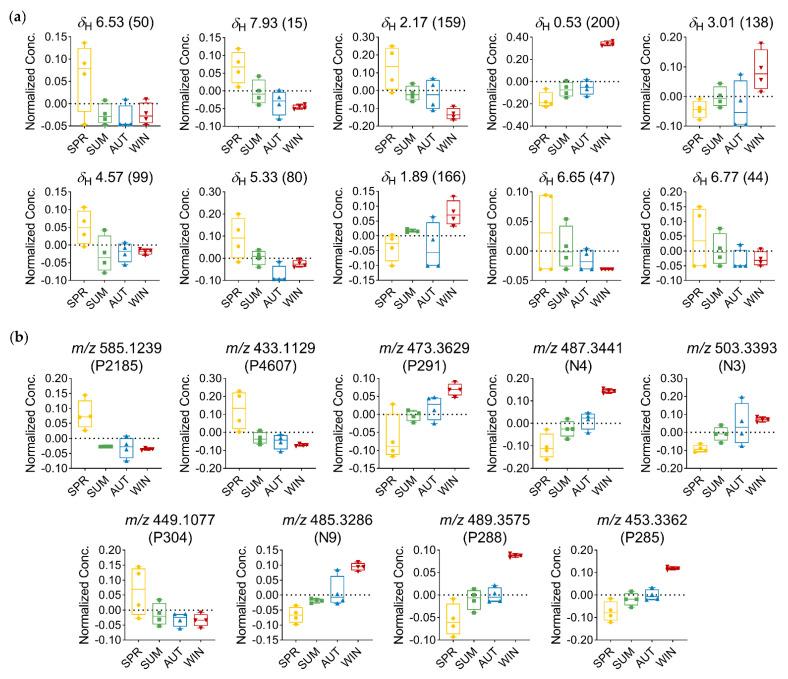
Relative distribution of discriminant metabolites in *T. catappa* extracts: (**a**) Box plots representing the concentration values of discriminant metabolites signals with FDR ≤ 0.05 (*δ* in ppm—ID) using MS-NMR fused data; (**b**) box plots representing the concentration values of discriminant metabolites signals with FDR ≤ 0.05 using MS data (*m*/*z*—MZmine ID). Season samples: Summer (SUM), Autumn (AUT), Winter (WIN), and Spring (SPR).

Based on the analysis of the relative distribution of the compounds in the *T. catappa* plant leaves, the box-and-whisker plots (Figure 6) showed that the signals related to aliphatic compounds, and annotated as being part of the triterpene structure, exhibited the highest concentration of these compounds during autumn–winter and the lowest concentration during spring. On the other hand, the protons of the aromatic region, which are attributed to the class of flavones, exhibited the highest concentration of these compounds in spring and the lowest in winter.

## 4. Discussion

The metabolomics analytical technique applied through the application of LC-HRMS and NMR spectral datasets was found to be fast and highly suitable for detecting possible seasonal variations in secondary metabolites present in the harvested plant species under study (Supplementary Figures S1–S3). It should be noted, however, that the analyses conducted under this approach usually generate a large data set, which often hinders a thorough assessment of all the information obtained. In this regard, the preprocessing procedure was essentially important for the data analysis (NMR and MS), as it allowed the removal of unwanted biases and experimental variations prior to performing the statistical analysis; as such, owing to the procedures employed, we were able to improve the quality of the signals and reduce noise interference, transforming the data into a robust compatible matrix of interpretable size [36,49,50,51].

Two-dimensional (2D) *J*-res NMR experiments have proven to be suitable for the metabolic study of complex plant mixtures. The separate acquisition of chemical shift data and spin–spin coupling in different axes reduced signal overlap in the spectra, and consequently allowed us to perform more accurate measurements [52]. In addition, *J*-res analysis made it possible to obtain information about the functional groups of certain classes of compounds, based on the chemical changes of structural characteristics and quantitative information, by integrating the signals with adequate resolution.

The application of the chromatographic method allowed a good separation of the main groups of metabolites, while the electrospray ionization technique (ESI) led to the formation of protonated and deprotonated molecules ([M+H]^+^ and [M-H]^−^). Moreover, depending on the ionization mode employed (negative or positive), certain classes of compounds exhibited greater sensitivity, as is the case of ellagitannins (Supplementary Figure S3, Rt ≈ 1–10 min), which displayed greater signal intensities in the negative mode. The carboxyl groups attached to these compounds endow them with an acidic character and make negative-mode ionization more feasible.

With regard to the metabolite profile, *T. catappa* extracts mainly exhibited compound groups of the class of hydrolyzable tannins (gallotannins and ellagitannins) glycosylated flavonoids, ellagic acid derivatives, and triterpenes (Supplementary Table S2). Gallotannins were found to be constituted by galloyl residues attached to a sugar moiety, which ranged from mono-*O*-galloylhexose (332 Da) to penta-*O*-galloylhexose (940 Da). The structure of the ellagitannins, on the other hand, was found to entail hexahydroxydiphenoyl (HHDP) groups [53]; these characteristics were observed for punicalagin anomers, punicalin, terflavin A, corilagin, chebulagic acid, and tellimagrandin I, among other ellagitannins.

The glycosylated flavonoid family was identified in three different groups: *C*-glycosylated, *O*-glycosylated, divided into mono- and diglycosylated, and *O*-galloyl flavonoids. Among the glycosylated flavonoids, the compounds with *m*/*z* 585.1239 [M+H]^+^ and *m*/*z* 601.1184 [M+H]^+^ are typical examples of *C*-glycoside flavone with galloyl substitution, which are characterized as vitexin and orientin derivatives, respectively [28].

The punicalagin and punicalin ellagitannins are considered chemotaxonomic markers of the *Terminalia* genus; these compounds have been found to possess pharmacological properties [54]. Punicalagin, for example, was found to exhibit antioxidant, anti-inflammatory, antigenotoxic, hepatoprotective, and antitumoral properties [55,56,57,58,59], among other properties.

With the application of multivariate analysis, we were able to visualize the influence of the changes that occur in the chemical composition of the samples as a result of seasonality. With regard to the region where the *T. catappa* species was harvested, the weather represented typical characteristics of a humid tropical coastal region. Summer periods are generally hot and humid, winter periods are characterized by milder temperatures and a lower incidence of rainfall, and spring and autumn periods are transitional seasons. Cold fronts are a very common phenomenon in this coastal region, and more precipitations are observed in the spring and summer periods.

Due to the transition months over the spring period, this season has shown to be the most scattered and was separated from the other seasonal groups. The spring season is intermediate between summer and winter, and its metabolic profile may be influenced by the environmental characteristics of late summer and early winter. This is clearly noticeable in the OPLS-DA plot (Figure 5a,c), where there is a separation of classes within the spring group, with samples harvested after winter in October (Tc5 and Tc12) (separated in a different group from those harvested before summer in November/December (Tc6 and Tc13). It should be noted, however, that the seasonal fluctuations in meteorological conditions do not seem to explain this distinctive grouping of the spring samples, such that the metabolic profile may have been influenced by time-based leaf growth/development stages. Furthermore, looking at the grouping pattern of the samples, the variation in the metabolism of the plant was regulated on the basis of the seasons of the year; this is parallel to the variations in the accumulation and production of metabolites, which followed the temporal trend of the seasons, varying from spring to winter.

With the identification of distinct groups, OPLS discriminant analysis allowed us to determine which variables led to the separation of the seasonal groups. Among the discriminant variables that were significant (FDR ≤ 0.05), the signals observed in the range *δ*_H_ 0.53–3.05 indicated the presence of three characteristic signals of methyl, methylene, and oxygenated methine protons of highly hydroxylated triterpenes compounds, which were attributed to the derivatives of oleanane (**N3**) and ursane (**P291**, **N4**, **N9, P288**, and **P285**) triterpenes [60], based on their mass spectral data (Table 1 and Table 2, Figure 5b,d). As for the variation in the amount of these metabolites, a progressive increase in the concentration of triterpene-related variables was observed between spring and winter, reaching maximum amounts in the winter season (Figure 6).

The increase in triterpene content during autumn and winter may be associated with the low levels of rainfall during this period. Moreover, the *T. catappa* plant species is a deciduous tree, and the leaves of the plant are known to fall during periods of drought, which is observed annually, and occurs in the winter season during the months of July and August. The falling of the plant leaves during this period is attributed to their senescence stage; this phase triggers many processes in the plant, which are mainly driven by regulatory networks responsible for the expression of hormones and the overproduction of reactive oxygen species (ROS) [61,62]. The plant regulators that participate in the initiation of the senescence process of the leaves are considered elicitors of many secondary metabolites in the plant, such as triterpenes [63,64,65]. In this case, the overexpression of these regulators during the senescence stage appears to have further induced triterpene biosynthesis in the plant, contributing to an increase in the concentration levels of these compounds during autumn/winter. In addition, some studies published in the literature have reported that pentacyclic triterpenes also play a role in plant defense against insects due to their antifeedant and phytotoxic effects [66,67]. 

Another relevant observation that is worth mentioning has to do with the chemical shift signals at *δ*_H_ 6.53, *δ*_H_ 7.93, *δ*_H_ 6.65, and *δ*_H_ 6.77, which are attributed to the signals in the aromatic proton region, found to be typically related to flavone-type flavonoids. The singlet at *δ*_H_ 6.53 is typically associated with H-8 when the A ring is substituted at position C-6; this is indicative of the compounds in which the A ring is tetrasubstituted. The singlet protons at *δ*_H_ 6.65 and *δ*_H_ 6.77 can be assigned to H-3 of the flavone nucleus; the chemical shift of these singlets may also correspond to the protons of a galloyl moiety. The signal at *δ*_H_ 7.94 (d, *J* = 8.8 Hz) is found to be typically associated with the B-ring protons of a flavone with ortho-coupling [28,68]. The doublet at *δ*_H_ 4.59 (d, *J* = 10.0 Hz) is assigned to the anomeric proton of a glucose moiety; this larger coupling constant is consistent with a *C*-glucoside in a *β* configuration [28,69]. In addition, the signal at *δ*_H_ 5.33 may also be attributed to the methine protons present in a saccharide unit. The chemical shift value indicates that these protons are deshielded, and this shows that the hydroxyl attached in the same position as the proton is acylated. Thus, in view of the data obtained, one can infer that these discriminant signals correspond to derivatives of flavones, which have glycosidic units and/or with galloyl substitution attached to them (Table 1, Figure 5b). Based on the discriminant features obtained from the MS data, these compounds can be identified as apigenin-6-*C*-(-*O*-galloyl)-hexose (**P2185**), apigenin-6-*C*-hexose (**P4607**), and luteolin-6-*C*-hexose (**P304**) (Table 2, Figure 5d). The maximum concentration of these metabolites was observed in spring, while the minimum concentration was observed in winter. Furthermore, the pattern of variation in *C*-glycosylated flavone derivatives was found to be quite opposite to that observed for triterpenes; this finding thus points to the fact that spring and winter are two distinct seasons (Figure 6).

Some studies reported in the literature have shown that leaves present a relatively higher content of phenolic compounds at the beginning of their development and growth, and this may explain the increase in *C*-glycosyl flavones observed in the *T. catappa* spring harvest; this phenomenon is attributed to the photoprotective function and protection against insect herbivory provided by these metabolites to the plant [70,71]. In addition, after a long dry period in autumn/winter, the rainfall rate increases in spring, allowing the plant to recover from water stress. Thus, as reported by Almeida et al. (2020) [72], 6-*C*-glycosyl flavones exhibit higher content during the rehydration phase, and therefore seem to contribute toward plant recovery. However, it is still unclear which mechanisms are responsible for the accumulation of *C*-glycosyl flavones by plant tissues in response to the recovery process.

Some other compounds, such as hydrolyzable tannins, may also play a role in plant defense. These compounds may contribute toward the protection of the plant against high temperatures and UV radiation, since light intensity strongly induces the biosynthesis of phenolic substances through the stimulation of various enzymes along the biosynthetic pathway [73]. The defense role played by hydrolyzable tannins may also be related to the protective mechanism of the plant against the extreme incidence of ultraviolet radiation. The ultraviolet absorption spectrum of ellagitannins shows absorption maxima of 218,260 and 379 nm [11]; this effectively indicates the capacity of these compounds to absorb UV radiation and to protect plant leaves from the damage caused by harmful UV-B radiation (280–315 nm) [74].

Under stress conditions, high temperatures, and solar radiation, plant metabolism experiences an imbalance between the formation and removal of ROS, and this leads to the accumulation of antioxidant compounds, which protect the plants from the damage caused by oxidative stress [75]. Thus, the relevant role played by antioxidant compounds in the plant defense mechanism against abiotic stress contributes to the production of phenolic compounds (e.g., hydrolyzable tannins and glycosylated flavonoids) in the plant during the stages of growth and development, which is mediated by their ability to scavenge ROS once they are generated.

## 5. Conclusions

This study provided relevant information on the metabolic profile of *T. catappa* and contributed to our understanding of the seasonal variation and the environmental conditions that interfere with its metabolic production, which may be useful to establish appropriate quality criteria for the standardization of medicinal plants.

The thorough metabolic analysis conducted in this study using mass spectrometry and nuclear magnetic resonance spectroscopy allowed us to characterize the metabolic profile of *T. catappa* leaf extracts. This metabolic analysis enabled us to identify hydrolyzable tannins, glycosylated flavonoids, and ellagic acid derivatives in *T. catappa* plant species. The application of data fusion in combination with multivariate analysis corroborated with the individual analyses, in terms of effectively identifying the metabolites that are responsible for the differences in chemical composition observed in the samples; this is because the combination of quantitative information on the NMR data along with the high resolution and precise mass of the MS allowed us to obtain more robust and reliable results.

Some differences observed in the metabolic profile may be related to the harvest period and the influence of environmental conditions (alternating periods between rainy and dry seasons); these factors may have affected the production and/or concentration of some secondary metabolites, which are mainly involved in the regulation of plant growth/development and senescence.

## Figures and Tables

**Table 1:** Discriminant features for the MS-NMR fused data that contribute to the differentiation of the seasonal harvests of *T. catappa* samples.

ID	*δ*_H_ (ppm) ^1^	*δ*_H_, Mult. (*J* in Hz)	Type of Proton	Annotation of Metabolite Class or Compound	*p*-Value	FDR ^2^
**50**	6.53–6.57	6.55, s	Aromatic, Ar-H	Flavones	0.00357	0.005
**15**	7.93–7.97	7.94, d (8.8)	Aromatic, Ar-H	Flavones	0.00358	0.010
**159**	2.17–2.21	2.19, t (7.4)	Alkyl (methylene), -CH_2_	NA	0.01110	0.015
**200**	0.53–0.57	0.55, s	Alkyl (methyl), -CH_3_	Triterpenes	0.01240	0.020
**138**	3.01–3.05	3.04, m	Alkyl (methine), -CH-OH	Triterpenes	0.01270	0.025
**99**	4.57–4.61	4.59, d (10.0)	Anomeric proton, -CH-OH	Saccharide unit	0.01940	0.030
**80**	5.33–5.37	5.34, m	Alkyl (methine), -CH-OH	Saccharide unit	0.02010	0.035
**166**	1.89–1.93	1.92, s	Alkyl (methine), -CH	Triterpenes	0.03480	0.040
**47**	6.65–6.69	6.67, s	Aromatic, Ar-H	Flavones/Galloyl group	0.04150	0.045
**44**	6.77–6.81	6.79, s	Aromatic, Ar-H	Flavones/Galloyl group	0.04400	0.050

^1^ Chemical shift range in the 1D projection spectra. ^2^ FDR: false discovery rate. s = singlet; d = doublet; t = triplet; m = multiplet; NA = not annotated. The splitting pattern has been suggested based on the result of *J*-resolved.

**Table 2:** Discriminant features for the MS data that contribute to the differentiation of the seasonal harvests of *T. catappa* samples.

MZmine ID ^1^	Rt (min)	Adduct (*m*/*z*)	Chemical Formula	Annotation of Metabolite Class or Compound	*p*-Value	FDR ^2^
**P2185**	8.42	585.1239 [M+H]^+^	C_28_H_24_O_14_	Apigenin-6-*C*-(-*O*-galloyl)-hexose	0.00005	0.006
**P4607**	7.67	433.1129 [M+H]^+^	C_21_H_20_O_10_	Apigenin-6-*C*-hexose (isovitexin)	0.00047	0.011
**P291**	22.92	473.3629 [M+H]^+^	C_30_H_48_O_4_	Hydroxyursolic acid	0.00581	0.017
**N4**	17.41	487.3441 [M-H]^−^	C_30_H_48_O_5_	Trihydroxyurs-12-en-28-oic acid (Asiatic acid)	0.00661	0.022
**N3**	15.67	503.3393 [M-H]^−^	C_30_H_48_O_6_	Tetrahydroxyolean-12-en-28-oic acid	0.00896	0.028
**P304**	6.74	449.1077 [M+H]^+^	C_21_H_20_O_11_	Luteolin-6-*C*-hexose (isoorientin)	0.00898	0.033
**N9**	16.50	485.3286 [M-H]^−^	C_30_H_46_O_5_	Trihydroxyursadien-28-oic acid	0.00976	0.039
**P288**	17.41	489.3575 [M+H]^+^	C_30_H_48_O_5_	Trihydroxyurs-12-en-28-oic acid (Asiatic acid)	0.01360	0.044
**P285**	17.42	453.3362 [M+H]^+^	C_30_H_44_O_3_	3-oxo-urs-12,18-dien-28-oic acid	0.01500	0.050

^1^ MZMine ID includes N = negative ionization polarity and P = positive ionization polarity. ^2^ FDR: false discovery rate.

## Data Availability

Not applicable.

## Supplementary Materials

The following supporting information can be downloaded at: https://www.mdpi.com/article/10.3390/metabo13030349/s1, Figure S1: One-dimensional *J*-resolved NMR spectra (DMSO-*d*_6_, 500 MHz) of *Terminalia catappa* samples (Tc1 to Tc13); Figure S2: Two-dimensional *J*-resolved NMR spectra (DMSO-d_6_, 500 MHz) of *Terminalia catappa* samples (Tc1 to Tc13); Figure S3: Base peak chromatogram for *Terminalia catappa* samples obtained from preprocessing by MZmine2: (a) negative ionization mode; (b) positive ionization mode; Figure S4: Permutation plot (n = 100 permutations) of PLS-DA validated model for *Terminalia catappa*: (a) NMR data; (b) MS data; (c) MS-NMR fused data; Figure S5: Permutation plot (n = 100 permutations) of OPLS-DA validated model for *Terminalia catappa*: (a) MS-NMR fused data; (b) MS data; Table S1: Weather characteristics for Santos-SP in 2017 and 2018: Average temperature, solar radiation, air humidity and rainfall; Table S2: Proposed compounds for seasonal harvests of *T. catappa* extracts by UHPLC-(ESI)-HRMS.
