# Supplementary material for: Understanding the Seasonal Effect of Metabolite Production in Terminalia catappa L. Leaves through a Concatenated MS- and NMR-Based Metabolomics Approach
Source: Metabolites. 2023 Feb 27;13(3):349. doi: 10.3390/metabo13030349 (PMC10053923; doi:10.3390/metabo13030349)
Supplement: Supplementary file 1 [file metabolites-13-00349-s001.zip › ACZ_Supplementary_material.pdf]

Supplementary Material

Understanding the seasonal effect of metabolite production in Terminalia catappa L. leaves through a concatenated MS- and NMR-based metabolomics approach

Ana C. Zanatta [1,2], Natália Carolina Vieira [1], Renato Dantas-Medeiros [3], Wagner Vilegas [2] and RuAngelie Edrada-Ebel [4,*]

[1] Department of Biochemistry and Organic Chemistry, Institute of Chemistry, São Paulo State University (UNESP), 14800-060 Araraquara, São Paulo, Brazil;
[2] Institute of Biosciences, São Paulo State University (UNESP), 11380-972 São Vicente, São Paulo, Brazil
[3] Department of Pharmaceutical Sciences, Faculty of Pharmacy, Federal University of Rio Grande do Norte, 59012-570 Natal, Rio Grande do Norte, Brazil
[4] Strathclyde Institute of Pharmacy and Biomedical Sciences, University of Strathclyde, G4 0RE Glasgow, Scotland, United Kingdom
* Correspondence: ruangelie.edrada-ebel@strath.ac.uk

Supplementary Content

Supplementary Figures **S2**
Figure S1. One-dimensional *J*-resolved NMR spectra (DMSO-d_6, 500 MHz) of *Terminalia catappa* samples (**Tc1** to **Tc13**) S2
Figure S2. Two-dimensional *J*-resolved NMR spectra (DMSO-d_6, 500 MHz) of *Terminalia catappa* samples (**Tc1** to **Tc13**) S3
Figure S3. Base peak chromatogram for *Terminalia catappa* samples obtained from pre-processing by MZmine2: (**a**) negative ionization mode; (**b**) positve ionization mode S4
Figure S4. Permutation plot (n = 100 permutations) of PLS-DA validated model for *Terminalia catappa*: (**a**) NMR data; (**b**) MS data; (**c**) MS-NMR fused data S5
Figure S5. Permutation plot (n = 100 permutations) of OPLS-DA validated model for *Terminalia catappa*: (**a**) MS-NMR fused data; (**b**) MS data S6
Supplementary Tables **S7**
Table S1. Weather characteristics for Santos-SP in 2017 and 2018: average temperature, solar radiation, air humidity and rainfall S7

Supplementary Figures

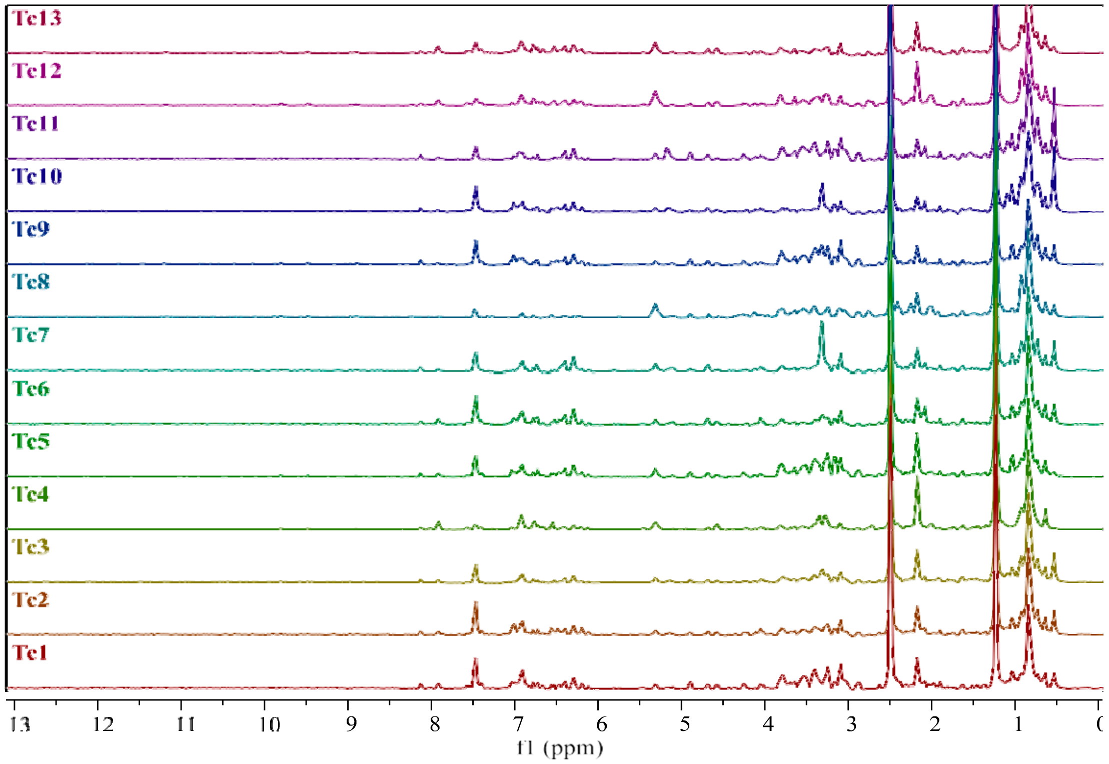

Figure S1. One-dimensional *J*-resolved NMR spectra (DMSO-d_6, 500 MHz) of *Terminalia catappa* samples (**Tc1** to **Tc13**).

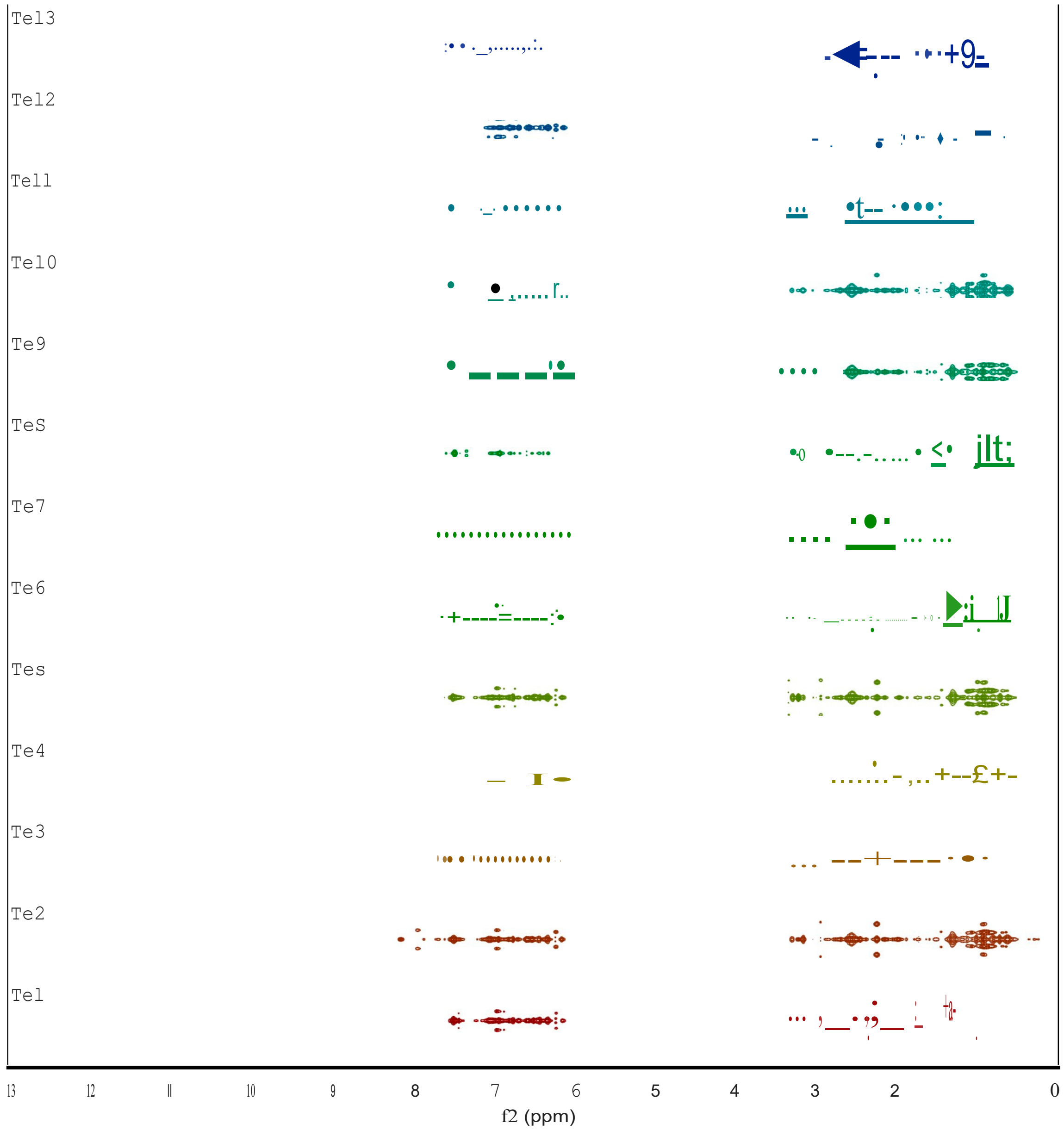

Figure S2. Two-dimensional *J*-resolved NMR spectra (DMSO-d_6, 500 MHz) of *Terminalia catappa* samples (**Tc1** to **Tc13**).

(a)

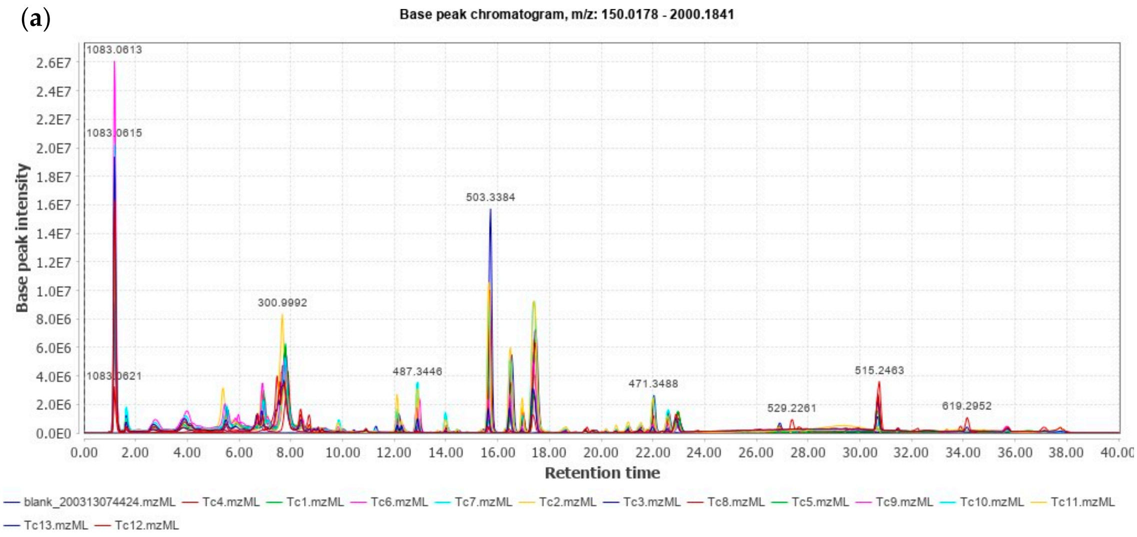

(b)

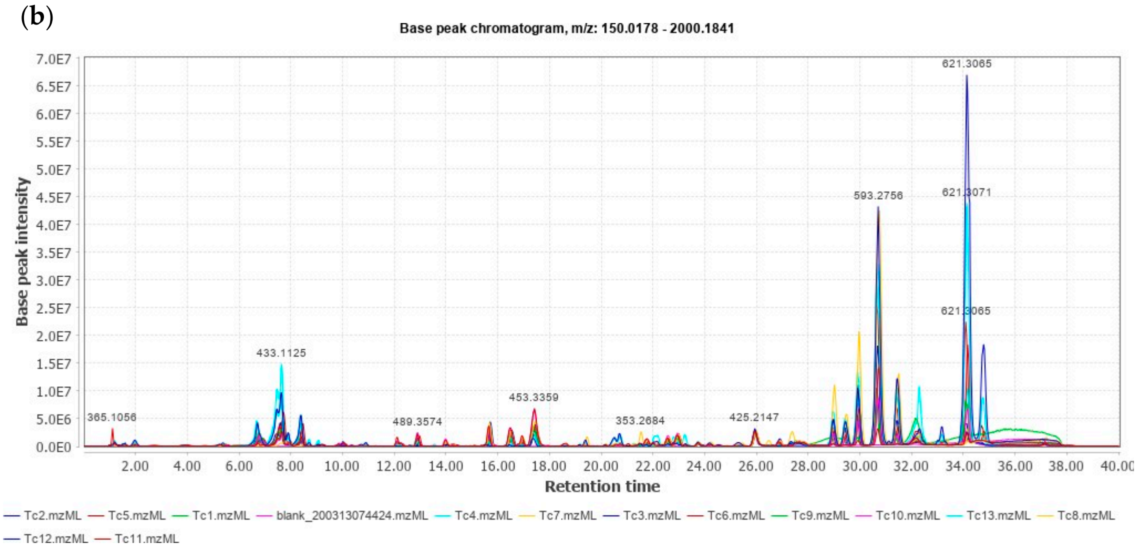

Figure S3. Base peak chromatogram for *Terminalia catappa* samples obtained from pre-processing by MZmine2: (**a**) negative ionization mode; (**b**) positve ionization mode.

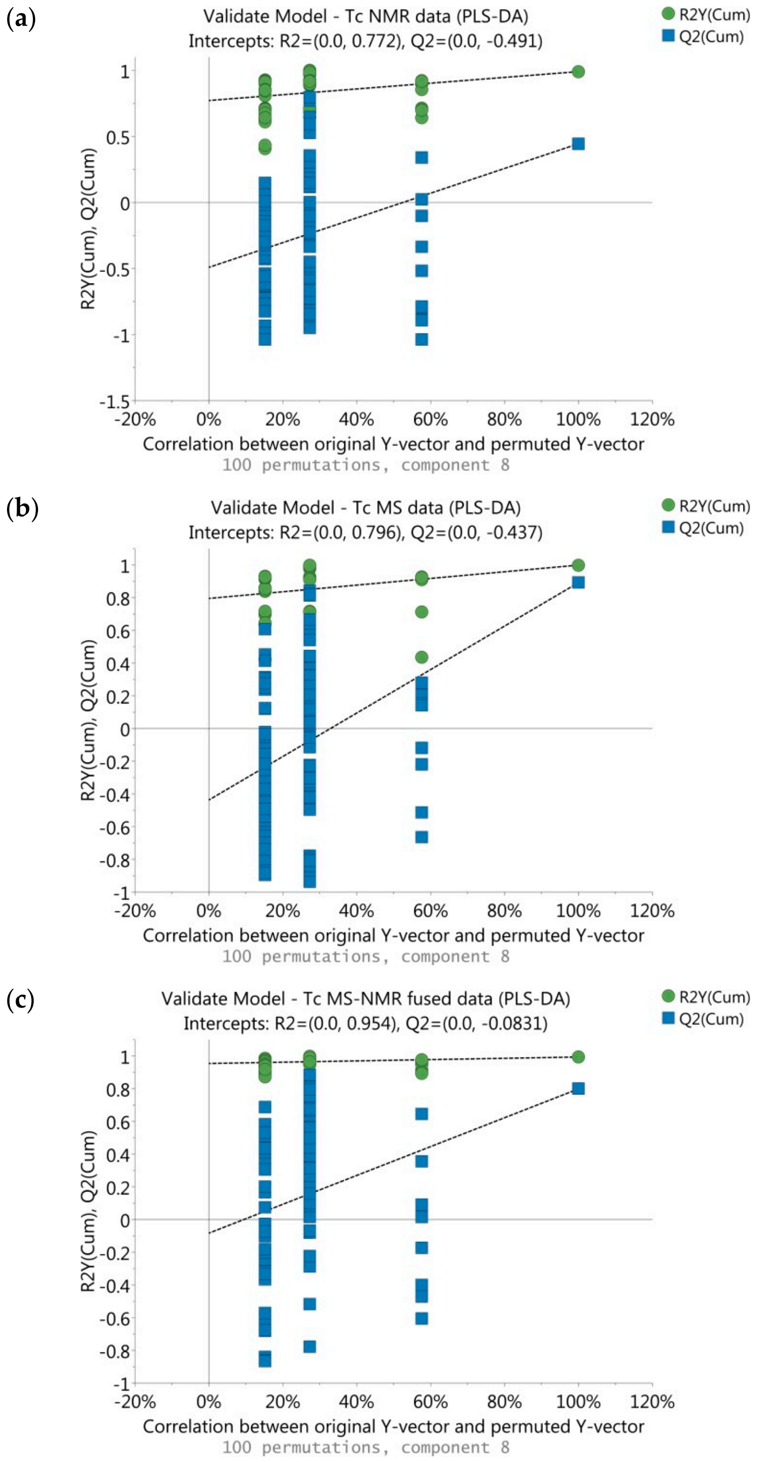

Figure S4. Permutation plot (n = 100 permutations) of PLS-DA validated model for *Terminalia catappa*: (**a**) NMR data; (**b**) MS data; (**c**) MS-NMR fused data.

(**a**)

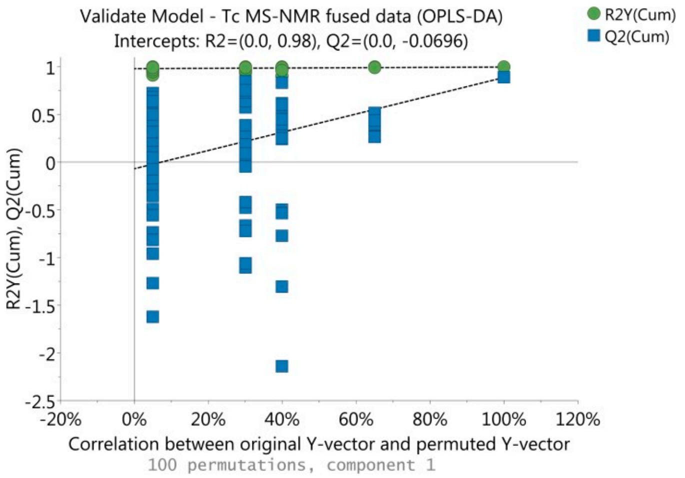

(**b**)

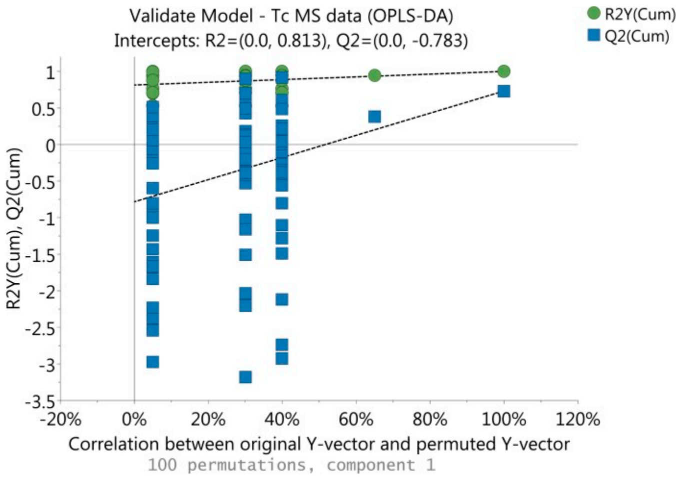

Figure S5. Permutation plot (n = 100 permutations) of OPLS-DA validated model for *Terminalia catappa*: (**a**) MS-NMR fused data; (**b**) MS data.

Supplementary Tables

Table S1. Weather characteristics for Santos-SP in 2017 and 2018: average temperature, solar radiation, air humidity and rainfall.

Period	Average of temperature (°C) per month	Average of solar radiation (kJ/m^2) per month	Average relative air humidity (%)	Cumulative amount of rainfall (mm) per month
Feb/17	29.4	2503.34	61	0.10
May/17	23.9	1551.68	70	0.19
Jun/17	23.1	1324.92	72	0.26
Aug/17	22.2	988.16	66	0.22
Oct/17	24.7	1079.44	75	0.19
Dec/17	25.2	771.04	84	0.43
Feb/18	30.4	901.93	75	0.60
Apr/18	27.4	931.46	70	0.38
Jun/18	24.6	1504.56	73	0.20
Jul/18	16.5	166.82	96	0.10
Aug/18	21.4	782.68	59	0.10
Oct/18	20.9	508.83	95	0.38
Nov/18	26.3	903.74	81	0.41

* The metereological data were provided by the Meteorological Data Storage Section (SADMET) of the National Institute of Meteorology (INMET). Accessed at: www.inmet.gov.br.
